# Coupling between beta band and high frequency oscillations as a clinically useful biomarker for DBS

**DOI:** 10.1038/s41531-024-00656-8

**Published:** 2024-02-21

**Authors:** Martina Bočková, Martin Lamoš, Jan Chrastina, Pavel Daniel, Silvia Kupcová, Ivo Říha, Lucia Šmahovská, Marek Baláž, Ivan Rektor

**Affiliations:** 1 Brain and Mind Research Program, Central European Institute of Technology, Masaryk University, Brno, Czech Republic; 2 First Department of Neurology, Masaryk University School of Medicine, St. Anne’s Hospital, Brno, Czech Republic; 3 Department of Neurosurgery, Masaryk University School of Medicine, St. Anne’s Hospital, Brno, Czech Republic; 4 Faculty of Medicine, Masaryk University, Brno, Czech Republic

**Keywords:** Parkinson's disease, Biomarkers

## Abstract

Beta hypersynchrony was recently introduced into clinical practice in Parkinson’s disease (PD) to identify the best stimulation contacts and for adaptive deep brain stimulation (aDBS) sensing. However, many other oscillopathies accompany the disease, and beta power sensing may not be optimal for all patients. The aim of this work was to study the potential clinical usefulness of beta power phase-amplitude coupling (PAC) with high frequency oscillations (HFOs). Subthalamic nucleus (STN) local field potentials (LFPs) from externalized DBS electrodes were recorded and analyzed in PD patients (*n* = 19). Beta power and HFOs were evaluated in a resting-state condition; PAC was then studied and compared with the electrode contact positions, structural connectivity, and medication state. Beta-HFO PAC (mainly in the 200–500 Hz range) was observed in all subjects. PAC was detectable more specifically in the motor part of the STN compared to beta power and HFOs. Moreover, the presence of PAC better corresponds to the stimulation setup based on the clinical effect. PAC is also sensitive to the laterality of symptoms and dopaminergic therapy, where the greater PAC cluster reflects the more affected side and medication “off” state. Coupling between beta power and HFOs is known to be a correlate of the PD “off” state. Beta-HFO PAC seems to be more sensitive than beta power itself and could be more helpful in the selection of the best clinical stimulation contact and probably also as a potential future input signal for aDBS.

## Introduction

Beta power hypersynchrony (13–35 Hz) in motor circuits has been a well-known pathophysiological marker of hypokinesia and rigidity in Parkinson’s disease (PD), correlating with the severity of these main motor symptoms and suppressible by dopaminergic treatment as well as by deep brain stimulation (DBS)^1–4^. A novel therapeutic approach called adaptive deep brain stimulation (aDBS), based on this main parkinsonian state biomarker, has been successfully introduced into clinical practice^5–7^. Together with this new technique based on local field potential (LFP) sensing, a new type of DBS electrode have been used, called “directional leads,” with segmented contacts making it possible to more precisely focus the volume of tissue activated within the targeted structure^8,9^. However, there are still some points and limitations to be addressed.

There is an individual oscillatory reactivity related to DBS. Frequency peaks in the STN vary among subjects. Some patients do not demonstrate beta power reduction as a response to DBS^10^. Equally, PD is a heterogeneous disease with differing severity of main motor symptoms and varying numbers of non-motor symptoms. Borderline phenotypes, such as tremor-dominant (TD) and postural instability gait disorders (PIGD), with distinct patterns of progression can be distinguished^11^. It is known that aDBS based on beta power sensing is not optimal for all patients. Individual patient-specific PD neural markers sensitive to DBS therapy might be more suitable for aDBS due to symptom heterogeneity^12–14^. Many other oscillopathies linked to PD have been described, including low frequencies and gamma band power reduction and changes in high-frequency oscillations (HFOs)^14,15^. Pathological cross-frequency interactions between different frequency ranges have been shown to play an important pathophysiological role. Coupling between the phase of slow activities and the amplitude of fast frequencies, such as the phase amplitude coupling (PAC) between beta and HFOs, have been described as linked to PD main motor symptoms^16^. HFOs and their cross-frequency and PAC have the potential to provide new biomarkers with direct implications in novel DBS therapy strategies^15,17,18^.

Compared to the conventional DBS leads that were used for recordings in the majority of the patients in this study, as subject recruitment started before the new systems were commercially available in our country, the new directional leads have a higher number of contacts and stimulation setting options. The earlier programing approach based on best clinical effect testing is now more complicated and time consuming. LFP evaluation of the beta power amplitude has been recommended as a tool for clinical practice for predicting the most efficient stimulation contact^19^. However, as mentioned above, beta power does not seem to be optimal for all the patients and PD symptoms. A search for new and more specific biomarkers is therefore necessary.

The aim of this work was to study the occurrence of beta-HFO PAC and its potential use in clinical practice.

## Results

We analyzed resting-state STN LFPs in 19 PD patients in the immediate postoperative period. First, the beta power characteristics were evaluated. The PAC was then calculated with a focus on relations between beta and HFO. The measures were next compared to:

- the exact electrode position using the Lead-DBS software,
- later stimulation contacts with best clinical effect on PD motor symptoms, chosen after the standard clinical postoperative testing and initial setting,
- structural connectivity estimation based on the Human Connectome Project atlas.
- Finally, the effect of dopaminergic medication was evaluated.

Beta power characteristics are shown in Table 1. Typical beta power peaks were not clearly detectable in 9 patients. Among the other 10 patients, the power of the beta peak was higher in the more affected STN in 6 cases (*p* = 0.031, the paired Wilcoxon signed-rank test, data with not normal distribution tested by the Kolmogorov-Smirnov test). However, the effect of laterality was not significant across the whole patient group (*p* = 0.432).

**Table 1:** Beta parameters

ID	Beta peak			
	Left STN		Right STN	
	Frequency [Hz]	Normalized Power [-]	Frequency [Hz]	Normalized Power [-]
1	19.5	29	22.5	13
2	no specific peak		no specific peak	
3	no specific peak		no specific peak	
4	23	37	22.5	52
5	27.5	18	26.5	16
6	no specific peak		25	23
7	no specific peak		25.5	19
8	17.5	27	19.5	**54**
9	no specific peak		no specific peak	
10	26	14	24.5	**40**
11	23.5	**33**	14.5	28
12	30	**18**	28	11
13	18.5	30	13.5	101
14	no specific peak		23.5	7
15	15	87	16	**223**
16	no specific peak		no specific peak	
17	16.5	32	16	**72**
18	no specific peak		no specific peak	
19	no specific peak		no specific peak	

Resulting beta parameters for both left and right nuclei in all subjects: center frequency of the beta peak and its power (concordance with the more affected side in bold). No specific peak states for non-conclusive results of the beta power analysis, where no clear beta peak was expressed in the power spectrum.

We were able to detect beta-HFO PAC in all of our patients. PAC was present in different HFO frequencies among the patients; moreover, there were lateralization differences within the same patients. This could be explained by the side difference in the severity of PD symptoms. Larger clusters of beta-HFO PAC were present in the more affected STN (*p* = 0.036, the paired Wilcoxon signed-rank test, data with not normal distribution tested by the Kolmogorov-Smirnov test) and the HFO frequency was lower than in the less affected STN (*p* = 0.024, paired the Wilcoxon signed-rank test, data with not normal distribution tested by the Kolmogorov-Smirnov test). Details are presented in Table 2.

**Table 2:** Beta-HFO PAC parameters

ID	Beta-HFO PAC dominant cluster					
	Left STN			Right STN		
	cluster size [-]	center beta frequency [Hz]	center HFO frequency [Hz]	cluster size [-]	center beta frequency [Hz]	center HFO frequency [Hz]
1	0.26	18	303	**0.47**	16	293
2	**0.05**	18	233	0.01	26	488
3	0.01	30	138	0.27	24	463
4	**0.2**	22	318	0.19	20	323
5	0.34	26	318	**0.39**	24	308
6	0.06	16	248	0.44	26	328
7	**0.27**	18	233	0.24	30	308
8	0.15	30	298	**0.16**	32	278
9	0.09	16	243	0.04	16	233
10	0.12	20	223	**0.38**	22	313
11	**0.4**	22	218	0.3	20	358
12	**0.25**	30	313	0.01	32	183
13	**0.45**	28	313	0.31	16	333
14	0.32	16	378	**0.44**	22	238
15	0.09	18	208	**0.37**	12	338
16	0.06	18	263	**0.08**	22	238
17	0.12	18	233	**0.37**	18	173
18	**0.25**	30	148	0.01	24	168
19	0.17	28	383	**0.21**	22	223

Resulting beta-HFO PAC parameters for both left and right nuclei in all subjects: size of dominant cluster (concordance with the more affected side in bold; the value represents the coverage of the region of interest in a PAC comodulogram; 0 – no coverage, 1 – full 100% coverage), center frequencies of dominant cluster in phase (beta) and amplitude (HFO).

### Beta-HFO PAC dependency on the lead location

**Fig. 1:**
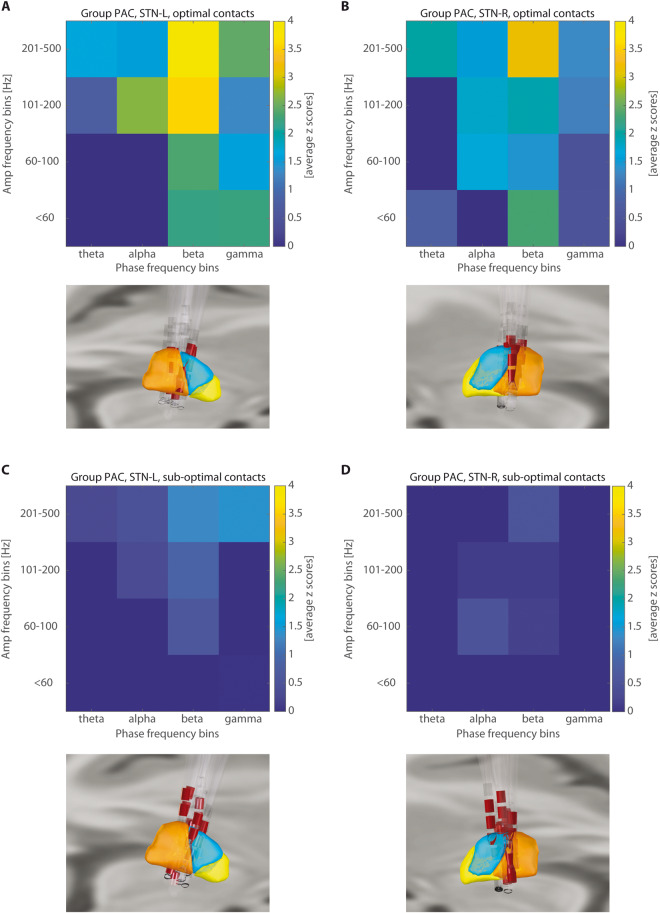
Group PAC pooled in frequency ranges with corresponding contact locations. **A**, **B** present contacts localized in the motor part of the left and right STN, noted as optimal. PAC is present bilaterally mainly in 200–500 HFOs range. **C**, **D** present contacts localized next to the motor part of the left and right STN, where no clear PAC is present, noted as suboptimal. In lead visualization orange color represents the motor part of the STN, blue is the associative part, and yellow corresponds to limbic part. Red color highlights contact pairs used for PAC calculation.

PAC was higher and more clearly detectable in the bipolar contact pairs localized within the motor part of the STN than in the other contacts (see Fig. 1). Contacts with the smallest distance to the STN motor part sweet spot (left STN = [−11 −14 −7] mm, right STN = [10.83 −13.31 −7.01] mm)^20^, were marked as optimal based on the lead localization approach. Other contacts were marked as suboptimal. The difference in the distance between the first and second closest contact to the sweet spot was significant across patients (left STN: *p* = 0.0001, right STN: *p* = 0.0001). Because the data did not have a normal distribution (tested by the Kolmogorov-Smirnov test), the paired Wilcoxon signed-rank test was used. The comparison between beta-HFO PAC clusters in optimal and suboptimal contacts was then assessed. The cluster size in the optimal contacts was significantly larger than the cluster size in the suboptimal contacts for both the left STN (*p* = 0.0005) and the right STN (*p* = 0.001). The cluster size variable did not have a normal distribution (tested by the Kolmogorov-Smirnov test) across patients; the paired Wilcoxon signed-rank test was therefore used.

### Beta power and beta-HFO PAC comparison for future best clinical contact selection

**Fig. 2:**
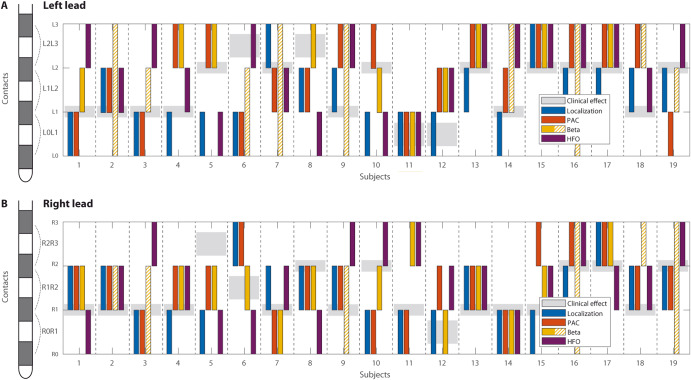
Comparison between PAC, beta power, HFOs. Comparison of the best contact selection according to: electrode localization by lead-DBS (blue), PAC (orange), beta power (yellow), and HFOs (violet). The gray background shows the contact selected according to the clinical effect. Yellow beta power bars where a clear beta peak in the spectrum was not detectable are shown hatched, see also lower contact specificity in these cases. **A** – left STN (contact pairs L0-L1, L1-L2 and L2-L3), (**B**) – right STN (contact pairs R0-R1, R1-R2 and R2-R3). In the context of electrode localization, PAC identified the optimal stimulation contact for the left STN in 7 of 19 cases (subject 1, 2, 3, 6, 8, 11, 15) and for the right STN in 13 of 19 cases (subject 1, 2, 3, 6, 8, 9, 10, 11, 13, 14, 17, 18, 19). Specific beta peak identified optimal contact in 2 cases for the left STN (subject 11, 15) and in 5 cases for the right STN (subject 1, 8, 13, 14, 17). In the context of the concordance with the clinical effect, PAC identified the optimal contact in 12 cases for the left STN (subject 1, 2, 3, 5, 7, 10, 11, 13, 14, 15, 16, 17) and in 14 cases for the right STN (subject 1, 2, 3, 4, 7, 8, 9, 11, 13, 14, 16, 17, 18, 19). Specific beta peak identified contact in 8 cases for the left STN (subject 1, 5, 8, 10, 11, 13, 15, 17) and 11 for the right STN (subject 1, 4, 6, 7, 8, 10, 12, 13, 14, 15, 17).

**Fig. 3:**
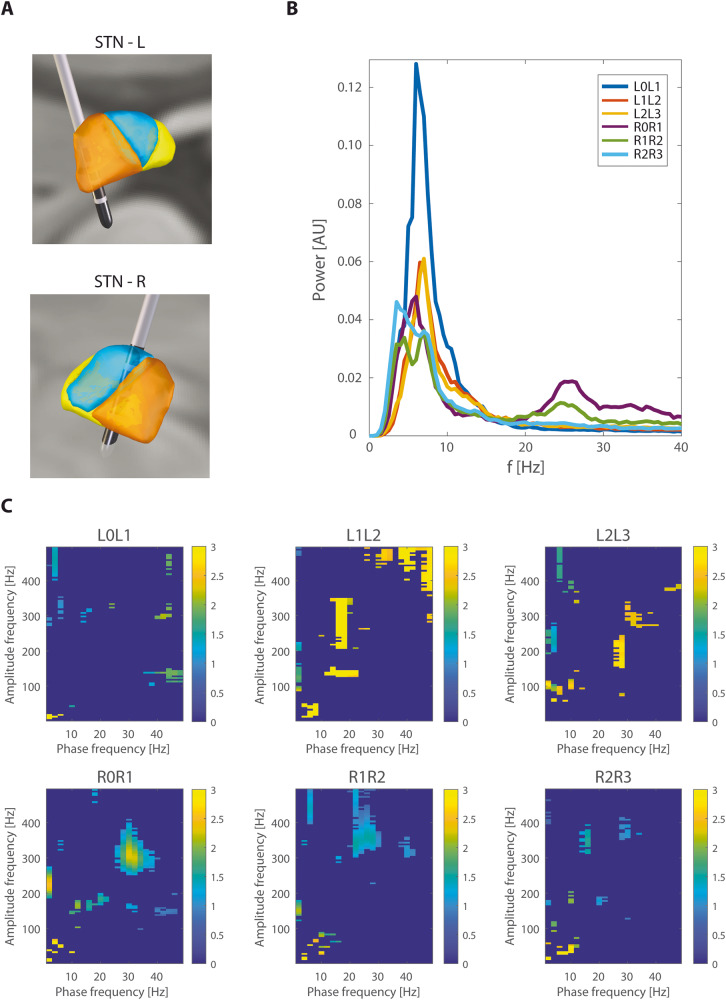
A case report example. Subject No. 7 (**A**) – electrode position reconstruction (orange – motor part, blue – associative part, yellow – limbic part of the STN), **B** – power spectrum, **C** – PAC. Beta power peak is sufficient for the right STN electrode but non-conclusive for the left STN, which is more severely affected. Here, the beta-HFO PAC clearly shows the bipolar pair L1-L2, which is best placed close to the motor part of the STN.

We evaluated beta power peak occurrence first. There were 9 cases for which beta power parameter was non-conclusive (see Table 1 and Fig. 2) because of no clear beta peaks present in the power spectrum. PAC seemed to be more specific for the motor part of the STN than the beta power enhancement itself, mostly in cases in which a clear beta peak was not present – see Figs. 2 and 3 for a case-based example. The stimulation contact selection based on beta-HFO PAC corresponded to the contact localization provided by Lead-DBS reconstruction in 7 cases in the left STN and 13 cases in the right STN. For beta power, there were only 2 cases in the left STN and 5 cases in the right STN. Tested by McNemar’s chi-square, beta-HFO PAC is significantly more accurate than beta power (*p* = 0.0003). The concordance with the contact selection based on the clinical effect was even higher, although it was not significant compared to beta power, McNemar’s chi-square, *p* = 0.09 (discussed in the study limitation section), 12 cases in the left STN and 14 cases in the right STN for beta-HFO PAC, and 8 cases in the left STN and 11 cases in the right STN for beta power (Fig. 2).

### Beta-HFO PAC and structural connectivity

**Fig. 4:**
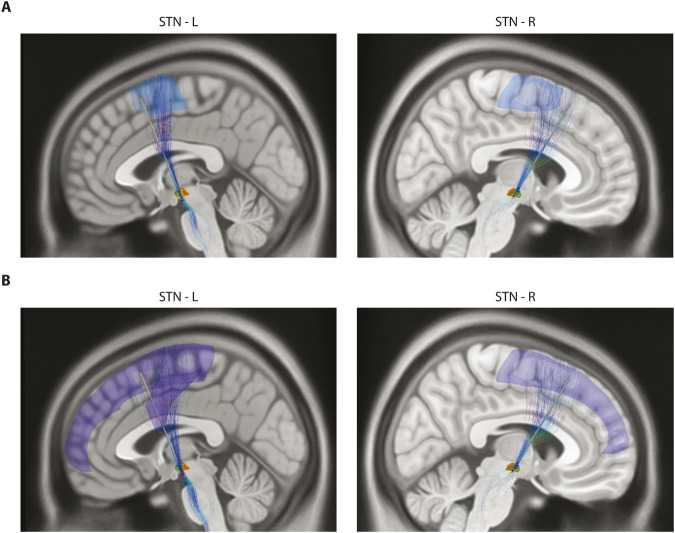
Structural connectivity. **A**– VTA from contacts with high beta-HFO PAC in the left and right STN connected to the SMA. **B**– VTA from contacts without beta-HFO PAC connected to wide fronto-parietal regions.

Structural connectivity was estimated using the Human Connectome Project^21,22^ for optimal and suboptimal contacts in each patient. This method has been described as having a predictive value similar to that of a patient-specific MRI tractography^21^. The activation of SMA in particular is thought to occur via the activation of fibers within the hyperdirect pathway and to have the highest importance for the DBS response in PD. In our study, the volume of tissue activated (VTA) created for contact pairs with the highest beta-HFO PAC (noted as optimal) were connected mainly to the supplementary motor area (SMA). VTA created for contact pairs with no or low beta-HFO PAC had structural connections to the SMA but also to other fronto-parietal structures – see Fig. 4. The activation of wide fronto-parietal areas is redundant and could also probably lead to the occurrence of adverse side effects.

### Beta-HFO PAC and dopaminergic therapy

In a subgroup of 10 patients, we had the possibility to study the influence of dopaminergic therapy; recordings were repeated after chronic medication intake, in the medication “on” state. In the other cases, this could not be done for technical reasons or due to patient fatigue.

Concerning the beta power analysis of left side STN, 6 patients had no clear beta peak and 4 patients had partial or total suppression of the whole beta peak by medication. For the right STN, there were 3 patients with no clear beta peak and 7 patients with partial or total suppression of the whole beta peak by medication – see Table 3.

**Table 3:** Effects of dopaminergic therapy

ID	Beta peak				Beta-HFO PAC	
	Left STN		Right STN		Left STN	Right STN
	Frequency [Hz]	Normalized Power [-]	Frequency [Hz]	Normalized Power [-]	Cluster size [-]	Cluster size [-]
1	no specific peak (29)		no specific peak (13)		0 (0.26)	0 (0.47)
2	no data		no data		no data	
3	no data		no data		no data	
4	no data		no data		no data	
5	no specific peak (18)		24	15 (16)	0.18 (0.34)	0.15 (0.39)
6	no specific peak (-)		no specific peak (23)		0.06 (0.06)	0.24 (0.44)
7	no specific peak (-)		25.5	18 (19)	0.04 (0.27)	0.02 (0.24)
8	no data		no data		no data	
9	no specific peak (-)		no specific peak (-)		0.01 (0.09)	0.02 (0.04)
10	no data		no data		no data	
11	no data		no data		no data	
12	no data		no data		no data	
13	18.5	20 (30)	14	56 (101)	0.01 (0.45)	0.14 (0.31)
14	no specific peak (-)		24.5	7 (7)	0.09 (0.32)	0.21 (0.44)
15	15	71 (87)	16	145 (223)	0.02 (0.09)	0.01 (0.37)
16	no specific peak (-)		no specific peak (-)		0 (0.06)	0.03 (0.08)
17	no data		no data		no data	
18	no data		no data		no data	
19	no specific peak (-)		no specific peak (-)		0.07 (0.17)	0.03 (0.21)

Beta power and beta-HFO PAC parameters influenced by dopaminergic therapy in a subgroup of patients. Results from medication “off” condition are shown in brackets. No specific peak description shows non-conclusive results of the beta power analysis, where no clear beta peak is expressed in the power spectrum.

**Fig. 5:**
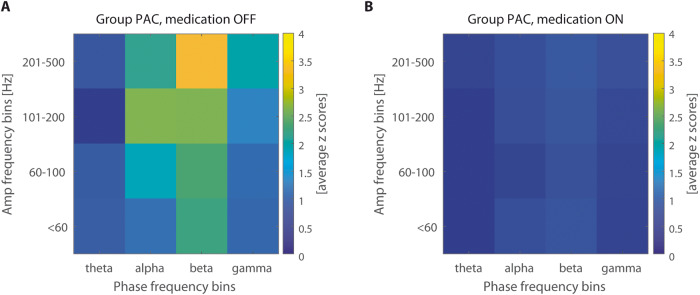
Group PAC pooled in frequency ranges influenced by dopaminergic therapy. **A** presents PAC in optimal contacts localized in pooled left and right STN, medication “off” state. **B** presents PAC in optimal contacts localized in pooled left and right STN, medication “on” state.

Beta-HFO PAC cluster size in the medication “on” state was significantly lower than the cluster size in the medication “off” state for the left STN (*p* = 0.004) and the right STN (*p* = 0.002); tested by the paired Wilcoxon signed-rank test – see Table 3, Fig. 5.

## Discussion

We recorded and studied intracranial data from the STN in PD patients in the immediate postoperative period following DBS surgery via externalized electrodes before the system internalization. This kind of research is challenging for the patients and clinical system; on the other hand, despite the epoch of sensing neurostimulators, it has the potential to provide new and useful information, mainly in the field of HFOs and their important impact.

The role of beta-HFO PAC in the pathophysiology of PD and its hypokinetic symptomatology has been described, as has its potential role in predicting effective DBS lead contacts to treat motor symptoms^15,23,24^. It has been shown that the origins of excessive beta power and HFOs are close but do not exactly overlap. The PAC relationship maximum is spatially localized more closely to HFO origins^24^. Therefore, localizing the best clinical contact based on beta power and PAC can differ and aDBS based on these different biomarkers could have different outcomes. Further studies with more detailed topographic mapping, imaging methods, and visualization techniques have been recommended to resolve these questions. We have therefore implemented retrospective electro-clinical correlations and new visualization methods, including in particular a structural connectivity evaluation based on the Human Connectome Project atlas^25^.

In this study, we confirmed the occurrence of beta-HFO PAC phenomena in the STN with the maximum in the motor part of the STN. In our group of patients, the beta power evaluation was sufficient for the clinical contact selection in only approximately half of the cases. In some cases or sides, the beta power analysis was not conclusive; beta peaks were not clearly expressed, mainly where the lead location was not optimal. In these lead contacts, PAC was detectable and correlated with the best contact placed in the motor STN according to the Lead-DBS tool and clinical setting based on the effect on motor symptoms. According to these analyses – see Fig. 2, beta-HFO PAC seems to be a more precise marker for selecting the stimulation sweet spot^26,27^.

Beta-HFO PAC is linked with beta power hypersynchrony, which is known to be a correlate of parkinsonian main motor symptoms and the “off” state condition. Pathological beta power probably impedes pro-kinetic HFO patterns when the beta-HFO PAC is prominent^24^. Beta burst dynamics vary during PD “off” and “on” clinical states. During “off” states, they have a long duration and strong bilateral interhemispheric phasic coupling^28^. In our study, we were able to confirm the reduction of the beta-HFO PAC during the “on” medication state, similar to the well-known beta power decrease after levodopa intake (see Table 3 and Fig. 5). Therefore, this PAC could also serve as a potential future input signal for aDBS, probably more specific than sensing based on beta power alone.

We observed interindividual variability in the frequency range of the beta-HFO PAC as well as lateralization differences in individual subjects. It is known that there are variations even in individual beta frequency peaks^29^. The beta-HFO PAC was more dominant in the more affected STN and shifted to lower HFO frequencies (Table 2). Weaker coupling and HFOs in higher ranges probably correspond with better parkinsonian states and vice versa.

Effective DBS treatment and good clinical outcomes are related to specific MRI structural connectivity that can be evaluated using human connectome data without the need for additional imaging^21^. The importance of anatomical connections of the DBS electrodes to the area of SMA has been confirmed^26^. In our study, we have documented that the electrode contacts with the highest beta-HFO PAC have structural connectivity only to the SMA, in contrast to the other contacts that can influence much wider frontal and parietal regions. From this point of view again, beta-HFO PAC seems to be a sensitive marker for optimal structural connectivity contact in the motor part of the STN with potentially the best clinical outcomes in PD therapy.

The main limitation of this study is that the recordings in the majority of our subjects were performed from older four-ring contact electrodes, as the data collection started at a time when the new directional leads were not yet available in our country. Monopolar signals from leads were recorded against scalp references; they had to be recalculated into a bipolar montage to evaluate LFPs in STN. Such a technical limitation can be confusing when compared with a clinical stimulation setting that is usually monopolar. These comparisons are presented in Fig. 2, where one of two neighboring bipolar contacts can be selected as a good match to a monopolar clinical setting.

Another limitation is that beta-HFO PAC seems to be more sensitive than beta power according to the postoperative clinical setting, but not significantly. However, the statistically significant difference is present in the context of concordance with lead localization.

Unfortunately, we were not able to correlate the PAC changes to the clinical state improvement after dopaminergic medication intake, as we could not administer the full MDS-UPDRS scale. Patients were on the first day post-surgery, not verticalized, and transported from the postoperative intensive care unit. MDS-UPDRS scores obtained at other time periods are not representative, as they could be influenced by many factors.

In conclusion, we were able to confirm the occurrence of beta-HFO PAC in all subjects. The main difference from previous studies focused on this topic is the direct comparison of beta-HFO PAC to beta power for identifying the optimal clinical contact for clinical practice using various types of approaches. In a subgroup of the patients, we confirmed the dopaminergic reactivity of the PAC phenomena. Greater PAC clusters were detected in the more affected STN. Beta-HFO PAC is probably more specific for the motor part of the STN. It could be a more useful biomarker for the best stimulation contact selection and probably also serve as a future input signal for aDBS. Further studies are necessary to confirm these findings.

## Methods

### Surgical procedure

The stimulation leads were implanted bilaterally into the STN by stereotaxic MRI-guided technique, including intraoperative microrecording and stimulation. After the implantation of both electrodes, a CT study was performed under stereotactic conditions covering the entire length of the implanted electrodes.

### Electrode contacts localization and connectivity

DBS electrode positions were reconstructed using Lead-DBS software (www.lead-dbs.org,^30^). Postoperative CT images were co-registered to preoperative MRI using advanced normalization tools (ANTs)^31^. Each step was manually checked in each patient. The structural connectivity from electrode contacts was estimated according to the anatomic connectome reconstruction based on the Human Connectome Project atlas, structural group connectome 32 Adult Diffusion MGH-USC HCP subjects GQI was used^21,22,25^. The seed areas were constructed as VTA by setting the stimulation parameters after the full course of clinical DBS optimization in each patient.

### Subjects, experimental protocol, and recordings

PD patients with externalized DBS electrodes implanted to the STN bilaterally (*n* = 19, see Table 4) in the immediate post-operative period (second day after surgery) before the system internalization participated in the study. Patients did not express signs of dementia or major depression and did not have any other clinically relevant severe problems according to detailed preoperative neuropsychological examinations. All subjects were informed about the scientific nature of this study and signed informed consent forms. The study received the approval of the local ethics committees (the ethics board of Masaryk University and the ethics board of St. Anne’s hospital in Brno). Five-minute resting-state LFP EEG was recorded from the STN in the medication “off” state, after 12 h of dopaminergic therapy withdrawal. In a subgroup of the patients (*n* = 10), the recording was repeated 45 min after chronic dopaminergic medication intake. Simultaneously, 128-channel scalp EEG were recorded. A few scalp contacts had to be blinded in each patient because of the sterile covering of the externalized DBS electrode area. The scalp data were used only for referencing the intracerebral contacts in this work. Subjects reclined comfortably in the monitoring bed, in a Faraday-shielded room. They were instructed to remain calm, to keep their eyes fixed on the monitor, and to avoid unnecessary movements. The sampling rate was 5 kHz, the recordings were monopolar with the average reference of all scalp electrodes, except blinded contacts and the most peripheral ones with a higher risk of possible muscle artifacts (F9, F10, FT9, FT10, T9, T10, TP9, TP10, P9, P10, Nz, Iz). The data were recorded by amplifier M&I BioSDA09 (M&I Ltd., Czech Republic) with internal sampling frequency 25 kHz/24 bit. For the 5 kHz output sampling rate, the maximal useful frequency bandwidth is 0.01–2000 Hz.

**Table 4:** Patient characteristics

ID	Sex	Age	Disease duration [years]	LED [mg]	Symptom severity dominant side	DBS system (IPG, leads)
1	M	63	16	1501	mild left side dominance	Activa, 3389
2	M	57	6	804	no clear side difference	Activa, 3389
3	M	62	7	1235	right	Activa, 3389
4	M	63	9	2008	right dominance of tremor	Activa, 3389
5	M	61	9	1861	mild left	Activa, 3389
6	M	54	6	1648	prominent right	Activa, 3389
7	M	67	12	1523	right	Infinity, 6146
8	M	62	9	998	left	Activa, 3389
9	M	64	5	1520	left	Activa, 3389
10	F	66	9	920	mild left	Percept, 3389
11	F	56	2	0	severe and drug resistant right side tremor dominant	Percept, 3389
12	M	57	8	1270	right, tremor dominant	Infinity, 6172
13	F	63	10	1137	right	Percept, 3389
14	M	62	8	1880	left	Percept, 3389
15	M	63	8	1208	left	Infinity, 6172
16	M	35	5	1880	left	Percept, B33005
17	F	50	7	1028	left	Infinity, 6172
18	M	62	7	1920	right	Infinity, 6172
19	M	50	9	1816	left	Infinity, 6172

*LED* levodopa dose equivalent, *IPG* implantable pulse generator.

### Signal preprocessing

The LFPs data were processed off-line using the EEGLAB toolbox^32^ and MATLAB 2021a (The MathWorks, Inc, Natick, USA). The signal was first filtered to 1–500 Hz by a second-order Butterworth filter. The filtration was performed in forward and reverse directions for zero phase distortion. A bipolar montage was performed to exclude the volume conduction from other structures and to confirm the local origin of the potentials^33,34^. It created three bipolar signals for the left STN marked as L0-L1, L1-L2, L2-L3 and similarly three bipolar signals for the right STN marked as R0-R1, R1-R2, R2-R3. Bad signals were detected and marked manually by visual inspection of the data in SignalPlant software^35^ and excluded from the subsequent analysis.

### Power analysis

For each bipolar signal (L0-L1, L1-L2, L2-L3 and R0-R1, R1-R2, R2-R3) the temporal fluctuations of beta power in the 12–30 Hz band and HFOs power in the 200–500 Hz band were computed by fast Fourier transform (FFT) in 10-second windows (Hamming) with 90% overlap and then averaged. To identify the dominant beta peak frequency, the power spectral density (PSD) estimate by periodogram was calculated with same window parameters and averaging.

### Beta-HFO PAC

The PAC was calculated using the modulation index (MI) method^36^. The MI approach generates a complex valued composite signal, where the amplitude is created from the high frequency amplitude envelope values and the phase matched to the low frequency signal’s instantaneous phase. The composite signal creates a joint probability density function in the complex plane. If the average of the signal is non-zero, it shows that a particular amplitude and phase value co-occur in time. An MI value then corresponds to the absolute value of the average of the composite signal.

Whole PAC computation was adopted from the PAC toolbox for MATLAB^37^. It uses shuffled datasets to evaluate the statistical significance of the calculated MI values^38,39^. The high-frequency amplitude signal was shuffled to disrupt the time-ordering of values. This was achieved by segmenting the data, the number of which was set equal to the number of seconds. The boundaries of the segments were placed at random locations chosen with uniform probability throughout the signal. The segments were randomly reordered to create a shuffled signal. The shuffling retained the mean, variance, and power spectrum of the original signal, whereas the temporal relationship between amplitude values is removed. Discontinuities are introduced and there is evidence that this can introduce spurious PAC detection results^38,39^; however, the performance on artificial data was still deemed sufficient, presumably since the discontinuities are independently distributed in time. A population of 50 shuffled signals were created and compared to the original low-frequency signal in order to generate a distribution of PAC values using the modulation index (MI) measure. PAC in the top 5% of the distribution were deemed significant. The resulting values were corrected for multiple comparisons by the false discovery rate (FDR). Patient-specific PAC comodulograms were constructed with significant relationships only.

PAC was computed for the phase 1–50 Hz (step 2 Hz) and amplitude 1–500 Hz (step 5 Hz). Filtering to predefined frequency bins was achieved via a convolution with complex Morlet wavelets with width = 7 (number of cycles defining the Morlet mother wavelet). Particular frequency bins of beta and HFO where coupling was presented were subject-specific and also side-specific (see Fig. 3). Beta-HFO PAC cluster size (Table 2) was evaluated as an area of the significant PAC presence in the window of phase 12–30 Hz and amplitude 200–500 Hz. Whole individual PAC comodulograms were then pooled in spectral ranges to show group results as a median across patients (Figs. 1 and 5).

Results of the PAC and power analysis were compared with symptom severity lateralization, contact positions within the STN using Lead-DBS software (Figs. 2 and 3, Tables 1 and 2), connectivity estimation using the Human Connectome Project atlas^25^ (Fig. 4), and with the consequent clinical effect based on postoperative clinical testing of each contact stimulation and clinical setting with the maximal improvement of motor symptoms without side effects after a full course of DBS optimization. Finally, the effect of dopaminergic therapy on beta power and beta-HFO PAC was examined in a subgroup of patients (Table 3 and Fig. 5).

### Reporting summary

Further information on research design is available in the Nature Research Reporting Summary linked to this article.

### Supplementary information

reporting summary

## Data Availability

The data supporting the findings of this study are not openly available. Anonymized data are, however, available from the corresponding author upon request.

## Code availability

The underlying code for this study is available from the corresponding author upon request.
