# Supplementary material for: Coupling between beta band and high frequency oscillations as a clinically useful biomarker for DBS
Source: NPJ Parkinsons Dis. 2024 Feb 21;10:40. doi: 10.1038/s41531-024-00656-8 (PMC10882016; doi:10.1038/s41531-024-00656-8)
Supplement: Supplementary file 1 — reporting summary [file 41531_2024_656_MOESM1_ESM.pdf]

nature portfolio

Corresponding author(s): Ivan Rektor

Last updated by author(s): Feb 6, 2024

Reporting Summary

Nature Portfolio wishes to improve the reproducibility of the work that we publish. This form provides structure for consistency and transparency in reporting. For further information on Nature Portfolio policies, see our Editorial Policies and the Editorial Policy Checklist.

Statistics

For all statistical analyses, confirm that the following items are present in the figure legend, table legend, main text, or Methods section.

n/a	Confirmed	
☐	☒	The exact sample size (n) for each experimental group/condition, given as a discrete number and unit of measurement
☐	☒	A statement on whether measurements were taken from distinct samples or whether the same sample was measured repeatedly
☐	☒	The statistical test(s) used AND whether they are one- or two-sided<br>*Only common tests should be described solely by name; describe more complex techniques in the Methods section.*
☒	☐	A description of all covariates tested
☐	☒	A description of any assumptions or corrections, such as tests of normality and adjustment for multiple comparisons
☐	☒	A full description of the statistical parameters including central tendency (e.g. means) or other basic estimates (e.g. regression coefficient) AND variation (e.g. standard deviation) or associated estimates of uncertainty (e.g. confidence intervals)
☐	☒	For null hypothesis testing, the test statistic (e.g. F, t, r) with confidence intervals, effect sizes, degrees of freedom and P value noted<br>*Give P values as exact values whenever suitable.*
☒	☐	For Bayesian analysis, information on the choice of priors and Markov chain Monte Carlo settings
☒	☐	For hierarchical and complex designs, identification of the appropriate level for tests and full reporting of outcomes
☒	☐	Estimates of effect sizes (e.g. Cohen's d, Pearson's r), indicating how they were calculated

Our web collection on statistics for biologists contains articles on many of the points above.

Software and code

Policy information about availability of computer code

Data collection	M&I BioSDA09 (M&I Ltd., Czech Republic)
Data analysis	MATLAB R2021a, EEGLAB 14.1.1, SignalPlant 1.2.4.0, PAC toolbox for MATLAB (https://data.mrc.ox.ac.uk/data-set/matlab-toolbox-estimating-phase-amplitude-coupling), Lead-DBS 2.5

For manuscripts utilizing custom algorithms or software that are central to the research but not yet described in published literature, software must be made available to editors and reviewers. We strongly encourage code deposition in a community repository (e.g. GitHub). See the Nature Portfolio guidelines for submitting code & software for further information.

Data

Policy information about availability of data

All manuscripts must include a data availability statement. This statement should provide the following information, where applicable:

- Accession codes, unique identifiers, or web links for publicly available datasets
- A description of any restrictions on data availability
- For clinical datasets or third party data, please ensure that the statement adheres to our policy

The data that support the findings of this study are available from the corresponding author upon the request.

Research involving human participants, their data, or biological material

Policy information about studies with human participants or human data. See also policy information about sex, gender (identity/presentation), and sexual orientation and race, ethnicity and racism.

Reporting on sex and gender	Parkinson's disease patients (n=19), 15 M, 4 F. Gender information was not collected. Not relevant in the study.
Reporting on race, ethnicity, or other socially relevant groupings	Race and ethnicity information was not collected. Not relevant in the study.
Population characteristics	Parkinson's disease patients with externalized DBS electrodes implanted to the STN bilaterally in the immediate post-operative period (second day after surgery) before the system internalization participated in the study. Patients did not express signs of dementia or major depression and did not have any other clinically relevant severe problems according to preoperative detailed neuropsychological examinations.
Recruitment	Movement Disorders Center, First Department of Neurology, Masaryk University School of Medicine, St. Anne's Hospital, Brno, Czech Republic
Ethics oversight	The Research Ethics Committee, Masaryk University

Note that full information on the approval of the study protocol must also be provided in the manuscript.

Field-specific reporting

Please select the one below that is the best fit for your research. If you are not sure, read the appropriate sections before making your selection.

☒ Life sciences ☐ Behavioural & social sciences ☐ Ecological, evolutionary & environmental sciences

For a reference copy of the document with all sections, see nature.com/documents/nr-reporting-summary-flat.pdf

Life sciences study design

All studies must disclose on these points even when the disclosure is negative.

Sample size	PD patients with externalized DBS electrodes implanted to the STN bilaterally (n=19). For medication ON analysis subgroup was used (n=10).
Data exclusions	artifacts from tremor or abnormal movements
Replication	Repeated calculations. Revealed phase-amplitude couplings between beta and high frequency oscillations are stable and reproducible.
Randomization	Not relevant, patient group contains all examined PD patients indicated to STN-DBS treatment.
Blinding	Blinding was not relevant for this study.

Reporting for specific materials, systems and methods

We require information from authors about some types of materials, experimental systems and methods used in many studies. Here, indicate whether each material, system or method listed is relevant to your study. If you are not sure if a list item applies to your research, read the appropriate section before selecting a response.

Materials & experimental systems

n/a	Involved in the study	
☒	☐	Antibodies
☒	☐	Eukaryotic cell lines
☒	☐	Palaeontology and archaeology
☒	☐	Animals and other organisms
☒	☐	Clinical data
☒	☐	Dual use research of concern
☒	☐	Plants

Methods

n/a	Involved in the study	
☒	☐	ChIP-seq
☒	☐	Flow cytometry
☐	☒	MRI-based neuroimaging

Plants

Seed stocks	*Report on the source of all seed stocks or other plant material used. If applicable, state the seed stock centre and catalogue number. If plant specimens were collected from the field, describe the collection location, date and sampling procedures.*
Novel plant genotypes	*Describe the methods by which all novel plant genotypes were produced. This includes those generated by transgenic approaches, gene editing, chemical/radiation-based mutagenesis and hybridization. For transgenic lines, describe the transformation method, the number of independent lines analyzed and the generation upon which experiments were performed. For gene-edited lines, describe the editor used, the endogenous sequence targeted for editing, the targeting guide RNA sequence (if applicable) and how the editor was applied.*
Authentication	*Describe any authentication procedures for each seed stock used or novel genotype generated. Describe any experiments used to assess the effect of a mutation and, where applicable, how potential secondary effects (e.g. second site T-DNA insertions, mosiacism, off-target gene editing) were examined.*

Magnetic resonance imaging

Experimental design

Design type	Structural scan only
Design specifications	Structural scan only
Behavioral performance measures	Structural scan only

Acquisition

Imaging type(s)	Structural
Field strength	3T
Sequence & imaging parameters	T1-weighted MPRAGE, 1mm iso voxel
Area of acquisition	whole brain

Diffusion MRI ☐ Used ☒ Not used

Preprocessing

Preprocessing software	Lead-DBS 2.5
Normalization	Lead-DBS -> ANTs module -> nonlinear diffeomorphic normalization
Normalization template	ICBM152
Noise and artifact removal	na
Volume censoring	na

Statistical modeling & inference

Model type and settings	na, only structural vizualization
Effect(s) tested	na, only structural vizualization

Specify type of analysis: ☒ Whole brain ☐ ROI-based ☐ Both

Statistic type for inference (See Eklund et al. 2016)	na, only structural vizualization
Correction	na, only structural vizualization

Models & analysis

n/a	Involved in the study
☒	☐ Functional and/or effective connectivity
☒	☐ Graph analysis
☒	☐ Multivariate modeling or predictive analysis
